# Evaluation of the “Cognitive Health Across Settings” intervention by clinicians and caregivers

**DOI:** 10.1093/geroni/igaf122.2424

**Published:** 2025-12-31

**Authors:** Mariya Kovaleva

**Affiliations:** University of Nebraska Medical center, University of Nebraska Medical Center College of Nursing, Nebraska, United States

## Abstract

We created a 21-day synchronous/asynchronous transitional care intervention for caregivers of persons living with dementia (PLWD), titled “Cognitive Health Across Settings (CHASE).” CHASE’s purpose is to educate and support caregivers during post-hospitalization period to reduce post-discharge complications and preventable emergency department visits and re-hospitalizations. CHASE’s synchronous elements are: two 30-minute videoconferences with caregivers 1-7 and 14-21 days post-discharge, led by a registered nurse (RN), a social worker, and a pharmacist. Asynchronously, caregivers watch video lessons (selecting from a library of 50 lessons) and read caregiver manual. RN meets with caregivers before CHASE starts to assess their and PLWD’s needs and create a checklist with tasks advisable to complete. We enlisted 10 stakeholders to evaluate CHASE. These included: one former and two current caregivers (for their parents); one geriatrician; one pharmacist; two social workers; and three RNs. These stakeholders provided quantitative (surveys) and qualitative (comments, focus groups) feedback on CHASE. Stakeholders evaluated the intervention mostly favorably. Criticisms included: information that is too complex and too long, not practical for busy caregivers to work with, and more suitable for health professions students. Clinicians considered the content too complex and overwhelming for caregivers – but caregivers did not perceive it as such, appreciating redundancy. Clinicians perceived presentation of skilled nursing facility experience as excessively negative. Stakeholders advised providing a glossary in the manual with medical jargon and worksheets that caregivers can pull from the manual. We are currently using this feedback to edit video lessons and manual prior to offering CHASE to caregivers.

